# Competitive Interactions between Two Non-Native Species (*Alliaria petiolata* [M. Bieb.] Cavara & Grande and *Hesperis matronalis* L.) and a Native Species (*Ageratina altissima* [L.] R.M. King & H. Rob.)

**DOI:** 10.3390/plants11030374

**Published:** 2022-01-29

**Authors:** Kassandra R. Paulus, Jordan M. Marshall

**Affiliations:** 1 Department of Chemistry, Purdue University Fort Wayne, Fort Wayne, IN 46805, USA; pavlkr01@pfw.edu; 2 Department of Biological Sciences, Purdue University Fort Wayne, Fort Wayne, IN 46805, USA

**Keywords:** competition, white snakeroot, garlic mustard, dame’s rocket, Asteraceae, Brassicaceae, invasive

## Abstract

*Alliaria petiolata* and *Hesperis matronalis* are wide-ranging non-native species in North America. *Ageratina altissima* is native to North America but has become a concern as an invasive species in Asia. A replacement series experiment was established to quantify the competitive interactions between these three species and to rank their relative competitiveness with each other. We assessed leaf count, chlorophyll content, and aboveground biomass with comparisons between replacement series mixtures and competition species. Overall leaf count and aboveground biomass were greatest in *A. altissima* and chlorophyll content was lowest in *A. petiolata*. Chlorophyll content and aboveground biomass were lower for *A. altissima* in competition with *A. petiolata* compared to *H. matronalis*. Leaf count for *A. petiolata* was lower in competition with *A. altissima* compared to *H. matronalis*. Aboveground biomass for *H. matronalis* was lower in competition regardless of the species compared to monoculture. There were also negative trends in biomass for *A. petiolata* in competition with increasing neighbors. However, for *A. altissima*, the negative trend in biomass was with *A. petiolata*, *H. matronalis* did not negatively affect *A. altissima* biomass. Our rank order of competitiveness was *A. altissima* > *A. petiolata* >> *H. matronalis*.

## 1. Introduction

Competitive ability and impacts on native communities, especially biomass accumulation, are important characteristics to understanding overall invasiveness of a given species [1]. Conversely, the existing community of plants has influence on successful establishment, overcoming the lag between cryptic and apparent, and subsequent dominance of non-native species invading an ecosystem. Community inertia may aid in reducing success rates of novel introduced species [2]. However, when population inertia from the non-native species is high due to adaptations overcoming establishment hurdles (i.e., non-viable offspring) and Allee effects (i.e., failure to locate mates), the likelihood of invasion outweighs the likelihood of exclusion [3]. Those traits that facilitate establishment may translate to competitive ability. Overcoming limitations in light [4,5,6], water [7], and nutrients [8,9], have implications on establishment and subsequent competition, often with benefits derived from phenotypic plasticity. Other traits add competitive advantage, such as early leaf phenology, seed production and dispersal, allelopathy, and biomass, leading to interrelated characteristics improving competitive success [10].

As a basic ecological interaction, competition results in negative influence on both individuals involved. However, improved competitive abilities can be a key mechanism for non-native species success and subsequent community level impacts related to invasion [11]. Even with similar traits, non-native species may be successful because they are better (i.e., faster, more efficient) at extracting resources compared to native species with which they are competing [12]. There is evidence that this competitive advantage may not last and non-native species populations decline over time in abundance and biomass [13,14]. Even if this type of decline will occur, there is a benefit to understanding relative competitive abilities of non-native species, especially in ranking species for management decisions.

*Alliaria petiolata* (M. Bieb.) Cavara & Grande (garlic mustard, Brassicaceae) is a wide-ranging species likely introduced to North America in the mid-1800s from Europe [15,16]. *A. petiolata* is an obligate biennial, germinating in spring, subsequently overwintering as a leaf rosette, flowering late in the following spring, and dispersing seeds in mid- to late-summer [17]. Community changes accompanying invasion by *A. petiolata* include decreases in forest understory native plant species diversity and reduction in leaf litter arthropod richness [18,19]. Impacts by *A. petiolata* on neighboring plants may be the result of allelopathic activity by this species, however, the degree of impact from allelopathy may be less important compared to other interactions, such as competition [20]. Multiple introductions from multiple source populations added to a relatively high genetic diversity for *A. petiolata* [21]. Additionally, *A. petiolata* is self-compatible with the capability of self-pollinating in closed flowers and produces thousands of seeds per individual [22]. A genetic pool sourced from different locations in the native range and self-compatibility leads to a high population inertia facilitating invasion, which adds to the complexity of control [23,24].

*Hesperis matronalis* L. (dame’s rocket, Brassicaceae) has a larger geographic range in North America compared to *A. petiolata*, despite a similar introduction date in the mid-1800s [25]. *H. matronalis* is also a biennial but has the capacity to overwinter as a short-lived perennial [26]. Because it was commonly used in garden plantings, it is possible *H. matronalis* was introduced multiple times. However, it mostly likely was introduced from a subspecies in western Europe [25]. Removal of *H. matronalis* from invaded areas results in little change in herbaceous native species abundance [27], which suggests this species may have little impact related to invasion. Additionally, there is little evidence that this species has any allelopathic influence on neighboring plants [28]. Unlike *A. petiolata*, *H. matronalis* is self-incompatible requiring insect pollination for reproductive success [29]. Introduction from a relatively limited native range and self-incompatibility would suggest that *H. matronalis* has low population inertia compared to *A. petiolata*. However, propagule pressure may be a sufficient force to overcome community inertia allowing for *H. matronalis* invasion [30].

*Ageratina altissima* (L.) R.M. King & H. Rob (white snakeroot, Asteraceae; syn. *Eupatorium rugosum* Houtt.) is a native species found in central and eastern North America [26]. *A. altissima* is a short-lived perennial, often associated with disturbance [31]. Large, wind dispersed seed crops and high germination rates likely has facilitated *A. altissima* as a non-native species invading South Korean forests [32,33]. Potentially, *A. altissima* has allelopathic capabilities [34]. In this non-native range, community inertia may be important in limiting invasion by *A. altissima* [35]. However, disturbance may facilitate *A. altissima* overcoming the invasion barrier [36].

**Figure 1:**
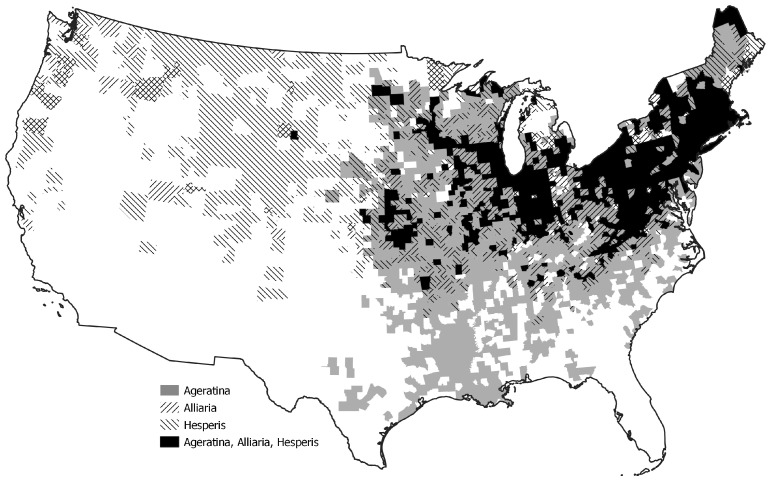
County distributions of *Ageratina altissima*, *Alliaria petiolata*, and *Hesperis matronalis* in the United States [37].

All three of these species can overcome invasion barriers in non-native ranges. Once established, it is important to gauge the relative competitive abilities to understand further mechanisms for success. The objectives of this study were to quantify the competitive interactions between two well established non-native species in North America and rank competitiveness of those two species using a commonly occurring native species. All three species co-occur in both geographic and habitat ranges in North America, specifically the United States (Figure 1). We were testing the hypothesis that in North America, the two non-native species have greater competitive abilities compared to the native species.

## 2. Results

**Figure 2:**
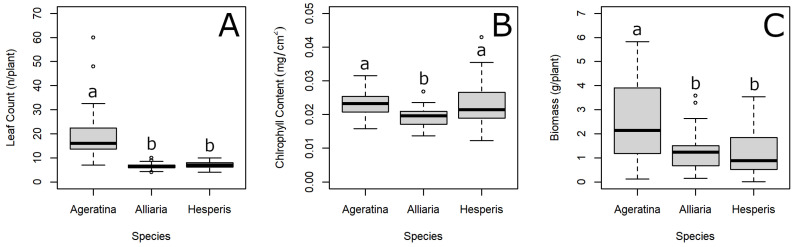
Leaf count (**A**), chlorophyll content (**B**), and biomass (**C**) comparisons between *Ageratina altissima*, *Alliaria petiolata*, and *Hesperis matronalis*. Unique letters indicate significant difference between species using Tukey’s HSD with a Holm adjustment as a post-hoc test following mixed-effect analysis of variance with series mixture and competition species as random effects. Heavy horizontal line represents mean; lower and upper edges of the boxes represent quartiles 1 and 3, respectively; lower and upper whiskers represent minimum and maximum, respectively; and open circles represent “outlier” values.

At the conclusion of the experiment, leaves were counted, chlorophyll content was measured, and plants were harvested for drying. Overall, leaf count was significantly different between species (Wald chi-squared = 126.24, df = 2, *p* < 0.001), with *A. altissima* having the most leaves (Figure 2A). There was also a significant difference in chlorophyll content between species (Wald chi-squared = 29.27, df = 2, *p* < 0.001), with *A. petiolata* having the lowest chlorophyll content (Figure 2B). Similar to leaf count, biomass differed significantly between species (Wald chi-squared = 41.12, df = 2, *p* < 0.001) with *A. altissima* having the greatest mass (Figure 2C).

While we replaced seeds that failed to germinate during the first two weeks of the experiment, those individuals that died after the cutoff date were not replaced. Proportion of individuals that died of a species in each series failed to meet the assumption of normally distributed data for all three species and was treated with an arcsine square root transformation. Overall, there was no significant difference between the three species mortality with series mixtures as a random effect (competition species was omitted from the mixed-effect ANOVA as it had zero variance; Wald chi-squared = 5.76, df = 2, *p* = 0.056). Mortality for *A. altissima* was not different between series mixtures or between competition with *A. petiolata* and *H. matronalis* (Table 1). *A. petiolata* did have differences in series mixture mortality, but not between the competition species. The mixture mortality difference manifested as a significant interaction, as well (Table 1). One individual *A. petiolata* in competition with four from the other species (4:1) had significantly lower proportion of mortality than the monoculture (*p* = 0.027). Similar to *A. altissima*, there was no mortality differences for *H. matronalis* (Table 1). Mean mortality rates were 18% for *A. altissima*, 15% for *A. petiolata*, and 28% for *H. matronalis*.

**Figure 3:**
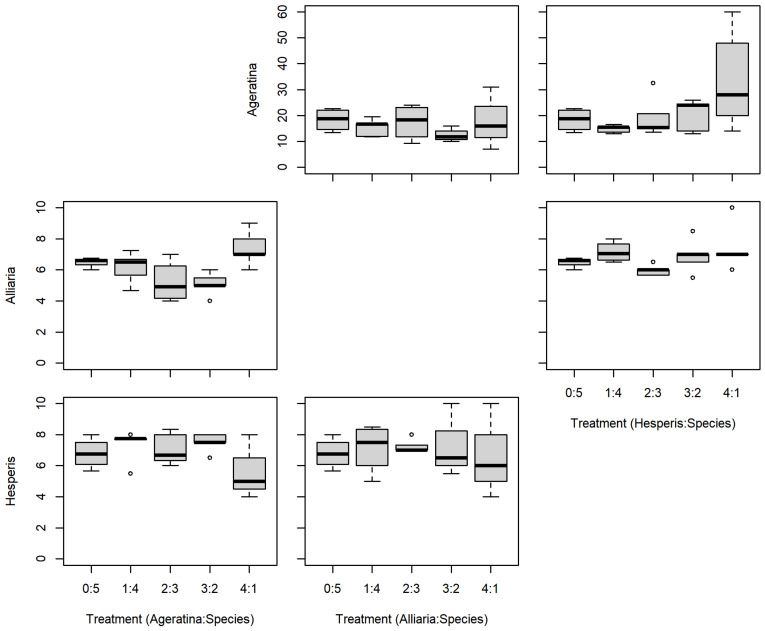
Replacement series leaf count results between *Ageratina altissima*, *Alliaria petiolata*, and *Hesperis matronalis*. Box and whisker symbols follow Figure 2.

**Figure 4:**
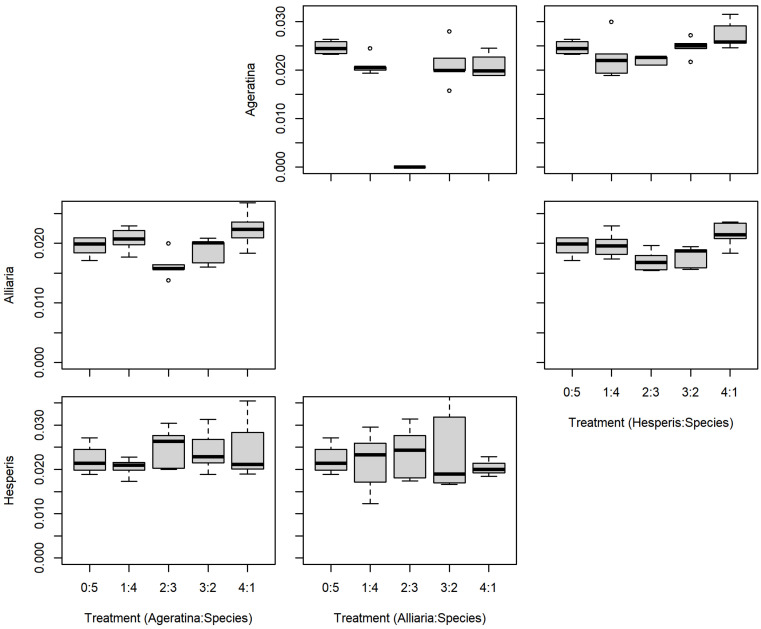
Replacement series chlorophyll content (mg/cm^2^) results between *Ageratina altissima*, *Alliaria petiolata*, and *Hesperis matronalis*. Box and whisker symbols follow Figure 2.

**Figure 5:**
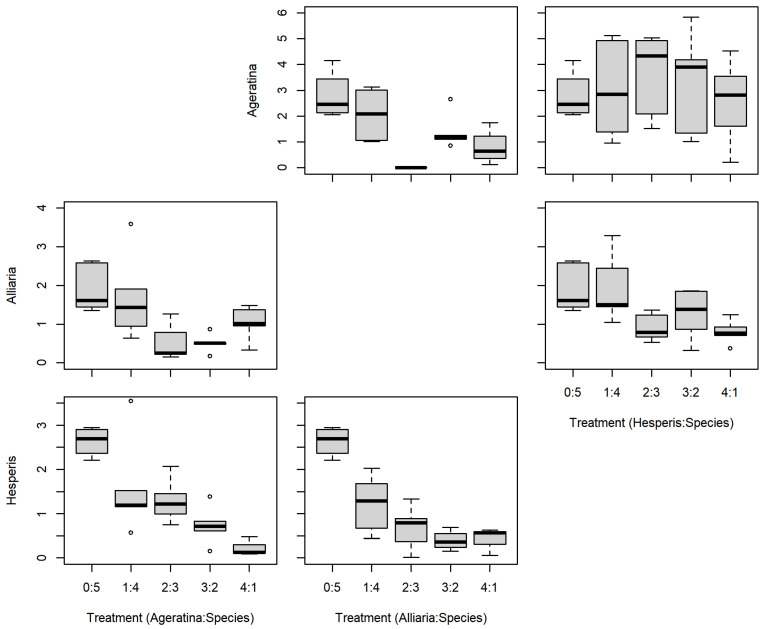
Replacement series aboveground biomass (g) results between *Ageratina altissima*, *Alliaria petiolata*, and *Hesperis matronalis*. Box and whisker symbols follow Figure 2.

There were no significant differences between the series mixtures and between competition species for *A. altissima* leaf count (Table 1; Figure 3). However, there was a significant difference in *A. altissima* chlorophyll content between competition species (Table 1; Figure 4); competition with *A. petiolata* resulted in reduced chlorophyll compared to competition with *H. matronalis* (*p* = 0.004). Similarly, *A. altissima* biomass did not differ between series mixtures, but was reduced in competition with *A. petiolata* (*p* = 0.022; Table 1; Figure 5).

For leaf count, *A. petiolata* experienced differences in mixtures and competition species (Table 1; Figure 3). While there was a significant F-value for the leaf count mixtures, none of those differences were with the monoculture (0:5). *A. petiolata* individuals in competition with *A. altissima* had reduced leaf number compared to those in with *H. matronalis* (*p* = 0.014). While there was a significant F-value for chlorophyll content (Table 1; Figure 4), none of the mixtures were different from the monoculture. *A. petiolata* biomass was different between the mixtures, but not between the competition species. Biomass in the 2:3 and 3:2 mixtures were less than the 0:5 monoculture (*p* = 0.005, 0.004; respectively; Figure 5).

There was no difference between series mixtures or competition species for *H. matronalis* leaf count and chlorophyll content (Table 1; Figure 3 and Figure 4). However, there was a significant difference in the series mixtures for biomass (Table 1; Figure 5). All of the series mixtures for *H. matronalis* had less biomass than the monoculture (1:4, *p* = 0.037; 2:3, *p* = 0.005; 3:2, *p* < 0.001; 4:1, *p* < 0.001).

Relative competitive intensity for *A. altissima* biomass was only positive for the 1:4 mixture with *A. petiolata* as competitor and negative for all mixtures with *H. matronalis* as competitor. Relative competitive intensity for *A. petiolata* was positive for the 3:2 and 2:3 mixtures with *A. altissima* as competitor and only for the 2:3 mixture with *H. matronalis* as competitor. Finally, relative competitive intensity for *H. matronalis* was positive for all mixtures with either *A. altissima* or *A. petiolata* as competitor. For relative competitive intensity, negative values represent a lack of competitive impact and positive values represent a competitive impact.

## 3. Discussion

*Alliaria petiolata* and *Hesperis matronalis* are common, wide-ranging, non-native species in North America [16]. While *Ageratina altissima* is native to North America, it was become a concern invading disturbed forests in South Korea [33]. Commonality and overlapping ranges of these three species was the impetus for this study to quantify competition and rank relative competitive abilities between *A. altissima*, *A. petiolata*, and *H. matronalis*.

Our mortality rates for *A. petiolata* were comparable to previous studies [38]. Similarly, our mortality rates for *H. matronalis* were also comparable to previous studies [38,39]. We were unable to locate seedling mortality values for *A. altissima* in greenhouse or garden experiments. In the one case of difference in mortality between a mixture (4:1) and the monoculture control for *A. petiolata*, it was the control that had increased mortality. From this and the literature, we conclude that none of these three species exert enough competitive influence at these densities to cause neighbor mortality.

Our results, specifically biomass, aligned with other competition studies including *A. petiolata* and *H. matronalis*. Reduction in *H. matronalis* biomass in competition with *A. petiolata* observed here has also been documented in other experimental designs [38]. The presence of neighboring plants has a strong negative influence on *H. matronalis* biomass [40]. Additionally, *A. petiolata* may be a strong competitor with other common forest species native to North America [41].

Even though they are confamilial and both considered invasive non-native species in North America, *H. matronalis* does not appear to have the same competitive abilities as *A. petiolata*. In our results, competition with *H. matronalis* did not influence mortality, leaf count, or chlorophyll content for the other two species. However, increasing density of *A. petiolata* did have a negative influence on *A. altissima* biomass. Interestingly, even though there may be biomass differences in competition with *A. petiolata*, removal of *A. petiolata* and *H. matronalis* from forests does not influence community richness or diversity [29,42].

Characteristics that make *A. altissima* a common native species in North America likely add to the species success as a non-native in Asia. There are potential allelopathic effects on neighboring plants with colonization by *A. altissima* [43,44]. Additionally, feeding on *A. altissima* can lead to toxicity in herbivores [45]. Combine these with a large wind dispersed seed crop, relatively high germination rates under a variety of storage conditions [46], and our competition results (i.e., decreased biomass for both *A. petiolata* and *H. matronalis*, reduced leaf count for *A. petiolata*), *A. altissima* is a strong competitor with the ability to dominate plant communities, especially with disturbance.

Relative competitive ability was not clearly arranged between these three species. This is a qualitative comparison of differences and patterns of results. *A. altissima* experienced significant reductions in chlorophyll content and biomass in competition with *A. petiolata* compared to competition with *H. matronalis*. For both measures, there was a negative trend when the numbers of *A. petiolata* increased. *A. petiolata* leaf count was the only significant difference between competition species, with reduced leaf count in competition with *A. altissima*. Because competition with *H. matronalis* did not result in significant reductions in leaf count, chlorophyll content, or biomass, we categorized it as the least competitive of the three species. This was also evidenced by the very clear negative trend in *H. matronalis* biomass with increasing individuals of the other two species. It is more difficult to separate *A. altissima* and *A. petiolata* regarding competitive ability. Patterns in biomass may help our differentiation of the species. *A. petiolata* had negative trends in biomass with increasing individuals of both *A. altissima* and *H. matronalis*. However, *A. altissima* only had that trend with *A. petiolata*. Because *H. matronalis* was able to influence the biomass patterns for *A. petiolata* but not *A. altissima*, we categorized the competitive ability of *A. altissima* above *A. petiolata*.

Both *A. altissima* and *A. petiolata* had positive relative competitive intensity values in mixtures together. Those positive values indicate that there was competitive impact from the competitor on the target species [47]. Conversely, negative values indicate a lack of competivie impact on the target species [47]. The difference for these two species was that *A. petiolata* had two mixtures with positive relative competitive intensity values in competition with *A. altissima*, while *A. altissima* had only one value that was positive in competition with *A. petiolata*. Since all relative competitive intensity values for *H. matronalis* were positive, then both *A. altissima* and *A. petiolata* had competitive impact on *H. matronalis*. Our final ranking of relative competitive ability between the three species was *A. altissima* > *A. petiolata* >> *H. matronalis*.

We were operating with *A. altissima* as a local, native species. It is common in its native range [26] and the competitive ability we demonstrate here likely aids in how common *A. altissima* is in North America. However, that competitive ability also adds to its abilities as an invasive species in Korea [33]. The ability to accumulate greater biomass compared to neighbors (e.g., note y-axis scales are larger for *A. altissima* leaf count and biomass in the above figures) has dramatic influence on the pre-emptive effect of invasion [35].

For the two non-native species, plasticity and response abilities to varying habitats may influence observed competitive abilities. Plasticity in habitat use (i.e., heavy shade and late-successional to more open habitats) found in *A. petiolata* likely results in a fairly competitive species [38]. That plasticity extends to energy allocation dependent on the light and water available in a habitat. Wetter sites with closed canopy lead to greater *A. petiolata* biomass allocation to leaves, stems, and roots, compared to drier sites with open canopy leading to greater biomass allocation to fruits and seeds [48]. Of the three species, *H. matronalis* was the least competitive. While it was least competitive here and removal of the species results in little community change [27], *H. matronalis* still invades edge habitats [25]. There may be site specific influences regarding the competitiveness of *H. matronalis* that may manifest in more mesic habitats [38].

Overall, *A. altissima* likely has greater competitive abilities compared to *A. petiolata* and *H. matronalis*. When the mixtures were random effects, *A. petiolata* and *H. matronalis* were significantly smaller individuals compared to *A. altissima* (both in leaf count and biomass). The negative trend in size for the two species in competition with *A. altissima* would likely have subsequent negative influence on second year growth and survival. Conversely, because *A. altissima* had significantly more leaves and more biomass in monocultures, reductions as a result of competition with *A. petiolata* would likely have relatively limited impact on second year growth and survival. A limitation to this interpretation is our study did not quantify flowering or seed production. Because *A. petiolata* and *H. matronalis* are biennial species, the second-year growth would be important to understanding population abundance dynamics as a result of competition with *A. altissima*.

## 4. Materials and Methods

### 4.1. Seed Collection and Preparation

Seeds for each species were collected from well-established populations at three sites in northeast Indiana, USA (41°07′06″ N, 85°05′44″ W; 41°13′47″ N, 85°51′27″ W; 41°14′03″ N, 85°52′30″ W), all associated with disturbance (i.e., along a recreation trail, along edges of managed forests). All seeds were collected between 29 September and 8 October 2020, and were cold stratified in wet sand at 4 °C from date of collection until sowing on 10 January 2021.

### 4.2. Study Design

A replacement series experimental design was used with five plants in each replicate pot. Seeds were sown in 10 × 10 cm pots in a sphagnum moss and perlite potting medium mixture. For our replacement series nomenclature, the first numeral represents the competitor and the second represents the target species (e.g., 3:2 with *Ageratina altissima* as the target and *Alliaria petiolata* as the competitor would include three individuals of the *A. petiolata* and two individuals of *A. altissima*). The inverse of a target and competitor were used to interpret the other species response (e.g., 3:2 with two *A. altissima* as target and *A. petiolata* as competitor was also interpreted as 2:3 with three *A. petiolata* as target and two *A. altissima* as competitor). This target and competitor nomenclature was our attempt at simplifying the interprtation of the results. Series pots were sown at ratios of monoculture (0:5, containing zero individuals of the other species and five individuals of the target species) and all combinations of 1:4, 2:3, 3:2, 4:1, for all three species. Each series included five replicates, for a total of 75 pots. Excess seeds were sown in greenhouse trays with the same potting medium and used to replace seeds that failed to germinate within the pots during the first two weeks. No seedlings were transplanted to replicates after the development of the first true leaf.

Pots were placed in an environment chamber room under cool white fluorescent light bulbs on a 16:8 day:night light cycle with daytime photosynthetically active radiation maintained at 1285 μmol/m^2^/sec (SD ± 418). Temperature was maintained at 22.2 °C (SD ± 0.4) with 20% relative humidity (SD ± 0.1). Series and replicates were randomly arranged under light fixtures and were randomly rearranged after eight weeks. Pots were watered as needed and were fertilized using a granular fertilizer (N-P-K 11-2-2) twice during the experiment—8 March and 5 April.

### 4.3. Data Measurement

The experiment was concluded when *A. altissima* individuals flowered (5 May 2021). At the completion of the study, leaf number per plant and leaf chlorophyll content were assessed for each individual. Our experimental unit was each pot, as such, leaf count was standardized per plant in each pot. The largest leaf per individual was selected and non-destructive chlorophyll content measurements were made with an atLeaf Chl meter (FT Green LLC, Wilmington, DE, USA). Meter values were converted to mg/cm^2^ chlorophyll content [49,50]. Aboveground plant material was harvested and dried at 50 °C, to a constant weight, with all individuals of a single species from a single pot pooled together. Similar to leaf count, biomass was standardized per plant in each pot. We calculated relative competitive intensity [47] with biomass as the measure of yield. Relative competitive abilities were categorically assigned to each species based on statistical analysis of mortality, leaf count, chlorophyll content, and biomass, as well as qualitative comparisons of patterns and relative competitive intensity.

### 4.4. Data Analysis

Overall comparisons between species for mortality, leaf count, chlorophyll content, and biomass were made using mixed-effect analysis of variance (ANOVA) with series mixture and competition species included as random effects. Tukey’s HSD (with a Holm adjustment) was used as a post-hoc test to identify differences between species. Leaf count, chlorophyll content, and biomass were compared between series mixtures and between competition species for our target species (e.g., *A. altissima* growth was compared between the series mixtures and between series in competition with *A. petiolata* and *H. matronalis*) using a two-way ANOVA. Dunnett’s test was used as a post-hoc to compare the target species growth in mixtures to the monoculture (0:5) as a control and Tukey’s HSD was used as a post-hoc test to compare the target species growth in competition with the two other species. Analyses were conducted in R using the *base* package, *lme4* and *car* packages for mixed-effect ANOVA, and *multcomp* package for Tukey’s HSD for the mixed-effect ANOVA and Dunnett’s test [51,52,53,54].

## Figures and Tables

**Table 1:** Two-way analysis of variance (ANOVA) results comparing *Ageratina altissima*, *Alliaria petiolata*, and *Hesperis matronalis* mortality, leaf count, chlorophyll content (mg/cm^2^), and biomass (g) in replacement series mixtures. Factor Series represents the mixtures for a species (0:5 as a monoculture of zero other species and five individuals, 1:4 as one individual of the other species and four individuals of the target species, 2:3, 3:2, and 4:1). Factor Competition represents competition with the other two species. Asterisk (*) indicates significant difference.

		Series				Competition				Interaction		
Species	Measure	df	F	*p*-Value		df	F	*p*-Value		df	F	*p*-Value
*Ageratina altissima*	Mortality	4, 40	1.26	0.303		1, 40	0.44	0.510		4, 40	0.37	0.827
	Leaf Count	4, 35	2.53	0.058		1, 35	4.06	0.052		4, 40	1.59	0.199
	Chlorophyll	4, 33	1.76	0.161		1, 33	10.47	0.003 *		4, 33	2.15	0.113
	Biomass	4, 33	1.44	0.244		1, 33	6.43	0.016 *		4, 33	0.77	0.517
*Alliaria petiolata*	Mortality	4, 40	6.96	<0.001 *		1, 40	0.69	0.413		4, 40	3.76	0.011 *
	Leaf Count	4, 38	4.91	0.003 *		1, 38	6.72	0.013 *		4, 38	1.61	0.191
	Chlorophyll	4, 40	8.67	<0.001 *		1, 40	0.47	0.498		4, 40	0.36	0.839
	Biomass	4, 40	7.94	<0.001 *		1, 40	1.57	0.217		4, 40	0.84	0.510
*Hesperis matronalis*	Mortality	4, 40	0.97	0.437		1, 40	0.26	0.613		4, 40	0.05	0.996
	Leaf Count	4, 33	0.79	0.541		1, 33	0.01	0.914		4, 33	0.26	0.900
	Chlorophyll	4, 33	0.50	0.734		1, 33	0.12	0.734		4, 33	0.24	0.914
	Biomass	4, 33	19.16	<0.001 *		1, 33	2.49	0.124		4, 33	0.60	0.664

## Data Availability

Data available in the Purdue University Research Repository at https://doi.org/10.4231/57AD-HR30 (accessed on 27 January 2022).
